# Network analysis of caffeine use disorder, withdrawal symptoms, and psychiatric symptoms

**DOI:** 10.1186/s12888-025-06478-z

**Published:** 2025-01-21

**Authors:** Mohammadreza Davoudi, Fatemeh Abdoli, Fereshte Momeni, Mojtaba Habibi Asgarabad

**Affiliations:** 1 Department of Clinical Psychology, School of Behavioral Sciences, University of Social Welfare and Rehabilitation Sciences, Tehran, Iran; 2 Department of Psychology, Norwegian University of Science and Technology, Trondheim, Norway

**Keywords:** Addiction, Caffeine, Caffeine use disorder, Network Analysis, Substance withdrawal syndrome

## Abstract

**Objective:**

Caffeine Use Disorder (CUD) is not currently recognized as a formal diagnosis in the Diagnostic and Statistical Manual of Mental Disorders, Fifth Edition (DSM-5). However, recent studies within the DSM-5 context have explored this issue. Also, this disorder is closely associated with caffeine withdrawal symptoms, which are formally recognized as a diagnosis in the DSM-5. Additionally, there is limited evidence regarding the connection between caffeine-related issues and psychiatric symptoms. The main aim of the present study was to determine the network structure of CUD and caffeine withdrawal symptoms among the general population. Also, the bridge symptoms among CUD, psychiatric symptoms, and caffeine withdrawal have been estimated.

**Method:**

Participants were 1228 adults (50.3% females, Mean age (x̄±sd) 35.49 ± 11.70 years) who completed Caffeine Use Disorder Questionnaire (CUDQ), Caffeine Withdrawal Symptoms Questionnaire (CWSQ), and Symptom Checklist-25 (SCL-25). All estimations were conducted according to the Gaussian Graphical Model.

**Results:**

“Excessive consumption” and “role obligations” were central symptoms in the CUD network. Difficulty in concentration was the most central node in the caffeine withdrawal network. Also, the obsessive-compulsive symptom emerged as a central and highly influential node in the relationship between caffeine-related nodes and psychiatric symptoms.

**Conclusions:**

Mental health providers should target these specific symptoms in clinical interventions to mitigate caffeine-related problems among individuals in the general population effectively.

**Supplementary Information:**

The online version contains supplementary material available at 10.1186/s12888-025-06478-z.

## Background

Caffeine, a natural psychostimulant alkaloid, can be found in over fifty plants, such as tea leaves, cocoa pods, and coffee beans [1, 2]. This psychoactive drug is identified as the most commonly consumed drug on the global scale [3]. According to the International Coffee Organization (2021), about nine million tons of coffee were consumed in various forms [4]. Caffeine consumption within recommended dietary guidelines is generally associated with positive outcomes, including heightened alertness and improved reaction times [5, 6]. However, DSM.5 encouraged evidence suggests that specific individuals may experience adverse health effects and functional impairment related to caffeine intake. These effects include elevated blood pressure (Derry et al., 2014), headache disorders [7], bone resorption [8], tinnitus [9], attention problems, and irregular heartbeat [10, 11].

Because there were so many problems related to excessive and maladaptive caffeine use and so much trouble with ingesting too much, the Diagnostic and Statistical Manual of Mental Disorders, Fifth Edition (DSM-5) presented two psychiatric disorders: Caffeine Use Disorder (CUD) and caffeine withdrawal. Caffeine withdrawal is a psychiatric disorder in the DSM-5, and CUD is still a new issue (Section III of the DSM-5) [12]. The CUD is a problematic pattern of caffeine use that leads to impairment or distress (i.e., symptoms of failure to quit, continued use in the face of illness, withdrawal, tolerance, craving, and disrupting everyday tasks). At least three of these symptoms must be observed during a 12-month period to be diagnosed [12]. As well, withdrawal can occur after long-term daily consumption of caffeine and abrupt decrease or discontinuation. In the space of 24 h, a headache, fatigue, mood disorder (irritability, depression), lack of concentration, or flu-like symptoms can emerge.

Several papers assessed whether these caffeine-related conditions were related to psychiatric symptoms. For instance, one paper assessed correlations between psychiatric symptoms (depression, anxiety, stress), caffeine withdrawal, and CUD [13]. These findings indicated that CUD severity was strongly correlated with caffeine intake. In addition, CUD was more likely to develop due to increased depression, anxiety, and stress. People with caffeine withdrawal symptoms had higher CUD and higher levels of depression, anxiety, and stress scores. Finally, the results showed significant associations between total caffeine intake and the level of CUD, depression, anxiety, and stress. Another study examined the relationship between caffeine and depression, anxiety, stress, and sleep in adolescents. The research revealed that caffeine consumption correlated with increased weight, lower performance at school, and scores on psychiatric symptoms such as depression, anxiety, insomnia severity, and the global measure of recent stress. Furthermore, those in the highest caffeine quartile showed a higher likelihood of severe insomnia even when controlling for potentially relevant psychological influences [14].

While recognizing the potential connection between caffeine and mental health issues, we must acknowledge that there are several research gaps here. Above all, studies of CUD and caffeine withdrawal, as described in the DSM-5, are relatively new. This beckons further investigation into these diseases to understand their cause and symptoms better. Although previous research has considered CUD and withdrawal holistically, it is imperative to continue exploring their symptomatology to gain a clearer understanding of their key features [15]. In addition, a handful of studies reported no significant correlation between caffeine and psychotic symptoms [16]. It’s time to address these research gaps better to understand the nuanced relationship between caffeine and mental well-being.

Network analysis is a novel way of diagnosing mental health and psychiatric disorders at the symptom level that aligns with the network theory of psychiatric diseases in depicting symptoms as interconnected nodes in a network. This strategy allows the definition of interrelationships between symptoms and core symptoms in the network using centrality markers such as betweenness, closeness, and strength [17]. Moreover, network analysis is increasingly used for its capacity to measure complex interactions between different clusters of symptoms within disorders [18]. This approach offers a holistic insight into symptom networks that extends beyond linear models to explain how symptoms are coupled to and influence each other networked [19].

To the best of our knowledge, no studies evaluated CUD and caffeine withdrawal at the network-level structure. Suggesting. This could potentially be tailored to treatments that refer to the most important symptoms and represent a new way to diagnose caffeine-related conditions within DSM-5. As far as we know, none of the network-based analyses of CUD and caffeine withdrawal have been performed. This would provide guidance on how to hone therapies by targeting the most important symptoms. Additionally, network analysis could provide a new perspective on diagnosing caffeine-related disorders within the DSM-5 framework.

### Present study

This study used a network approach to investigate the structure of CUD and caffeine withdrawal at the symptom level. By analyzing the interconnectedness of symptoms, we aimed to determine the underlying dynamics of these caffeine-related disorders at the symptom level. Accordingly, the current study aims were:

#### Estimate caffeine use disorder network structure

We aimed to model the connections between CUD symptoms using a network estimation. We tried to present the dominant symptoms at the disorder’s core by analyzing the network structure. These included desires or lack of control, use despite challenges, withdrawal, overuse, role responsibilities, social/interpersonal difficulties, tolerance, time spent on caffeine, and craving. We wanted to explain, through network analysis, how these symptoms influenced each other and what it might mean for CUD to happen.

#### Estimate the network structure of caffeine withdrawal symptoms

This goal was to develop a network model for explaining the structure of caffeine withdrawal symptoms. The analysis covered headache, fatigue, anxiety, depressed mood or irritability, poor focus, and flu-like symptoms. Through exploring the associations between these withdrawal symptoms, we aimed to find key symptoms that could influence the appearance and severity of caffeine withdrawal. This network-based understanding of caffeine withdrawal symptoms allowed targeted interventions to minimize withdrawal pain and improve outcomes in patients who were caffeine-dependent.

#### Evaluation of modal links between psychiatric symptoms and caffeine-induced symptoms

The goal was to investigate how psychiatric symptoms can impact caffeine-induced symptoms and vice versa. By exploring how symptoms of psychiatric conditions – depression, anxiety, stress – interact with symptoms of CUD and caffeine withdrawal, we aimed to find bidirectional pathways and common processes. This was essential to developing a multi-faceted model of how caffeine withdrawal symptoms could trigger psychiatric symptoms, and vice versa. Furthermore, it enabled us to see where we could target interventions and create more tailored treatment plans that addressed caffeine and psychiatric symptoms simultaneously.

### Method

All procedures adhered to the ethical guidelines outlined in the Helsinki Declaration. The research protocol (IR.USWR.REC.1401.037) was approved by the Ethics Committee of the University of Social Welfare and Rehabilitation Sciences (USWR), Tehran, Iran, in May 2022. This study is part of a national project examining patterns of caffeine use and problematic caffeine use in Iran (IR.USWR.REC.1401.037). The first paper from this project presented the prevalence of Caffeine Use Disorder (CUD) in an Iranian sample, and the current study represents the second paper derived from this larger project (please check [20]).

### Study design and participants

The study employed a cross-sectional design and was conducted in Tehran, Iran, from December 2022 to September 2023. Data collection involved administering a battery of paper-and-pencil questionnaires. Inclusion criteria comprised individuals who: (a) expressed interest in participating in research, (b) reported consuming at least one caffeine-containing substance at least 5 days per week, (c) were aged 18 or above, and (d) demonstrated proficiency in reading Farsi text. A total of 1,228 participants completed the survey for the study. As shown in Table 1, the respondents were predominantly young, with a mean age of 35.49, and nearly half were female (*n* = 618). For more information about the population, please check the mentioned paper in the first paragraph of the method.

**Table 1:** Demographic characteristics of the study sample

Variable		Mean (SD)/*n*(%)
Age		35.49 ± 11.70
CUD		7.18 ± 5.26
Somatization		4.90 ± 4.02
OCD		3.58 ± 2.65
Interpersonal Sensitivity		2.89 ± 2.50
Depression		1.98 ± 1.86
Anxiety		2.54 ± 2.63
Hostility		1.48 ± 1.65
Paranoid		.78 ± 1.06
Phobic		1.63 ± 1.80

### Procedures

First, a comprehensive recruitment announcement was crafted and disseminated online and in person across Tehran, Iran. This announcement was shared via popular social media platforms such as WhatsApp and Telegram, along with the distribution of Google Form links. The announcement provided a clear overview of the study’s objectives, assured participants of confidentiality, and delineated the procedure for participation. Participants were given information on the study tools: the CUD Questionnaire (CUDQ), the Caffeine Withdrawal Symptoms Questionnaire (CWSQ), the Symptom Checklist-25 (SCL-25), and the Demographic Information Questionnaire. They also provided a brief orientation to all participants (physically for paper-pencil participants and via Zoom or Google Meet for remote participants) to familiarize them with the study criteria.

Once volunteers assessed the inclusion criteria, they filled out these scales, which took about 10–15 min per person. Additionally, written informed consent was obtained from all participants before conducting the research. An attempt was made to structure the questionnaires for both paper-pencil and online versions of the study to minimize potential bias in data collection. More specifically, a counterbalancing strategy was employed to ensure that all subjects came across the questionnaires randomly. With each participant randomly assigned to a sequence of questions, any potential impact of the sequence in which the questions were administered was effectively minimized. This method tried to eliminate any possibility of order effects or sequence biases that might influence the results of respondents. Furthermore, by changing the order of the questionnaires from one participant to the next, the study aimed further to improve the internal validity of the data collection process. By adopting this counterbalancing process, the research sought to reinforce the validity of the collected data by increasing their strength and reliability. They also made sure that users could leave the project at any time without repercussions.

### Measures

***Caffeine Use Disorder Questionnaire (CUDQ)***: The CUDQ was a 9-item scale that was first developed to determine the level of caffeine symptoms experienced during 12 months [21]. To be consistent with the proposed DSM-5 diagnostic criteria, we re-designed the original Likert scale as a binary response (people could choose either “yes” or “no”) question. For CUD eligibility, patients had to verify at least three things about their caffeine consumption: (1) a recurring desire to cut back on caffeine use, (2) continued use even when the effects were negative, and (3) withdrawal symptoms. This Persian edition of CUDQ was also analyzed, and its scores exhibited good psychometric properties (Cronbach’s alpha = 0.77, Test-retest reliability = 0.94) [22]. In this research, Cronbach’s alpha was 0.75.

***The Caffeine Withdrawal Symptoms Questionnaire (CWSQ)*** was developed by Juliano et al. (2012) and assesses five withdrawal symptoms experienced within 24 h following a sudden reduction in caffeine intake. This scale included a binary response system based on “*Yes*” or “*No*” responses. The questionnaire has a Cronbach’s alpha of 0.78 [3]. The Persian version of the CUDQ also demonstrated satisfactory psychometric properties, with a reported test-retest reliability of 0.98 [22]. In the present study, the Cronbach’s alpha for the CWSQ was calculated as 0.66.

***Symptom Checklist-25 (SCL-25)*** was developed for the evaluation of psychiatric symptoms in the Iranian population. It is a 25-item questionnaire, a shorter version of the SCL-90, most of which are taken straight from the Hopkins Symptom Checklist. SCL-25 is a comprehensive psychological questionnaire that measures symptoms and distress in nine categories: physical complaints, obsessions, compulsions, interpersonal sensitivity, depression, anxiety, aggression, phobia, paranoid ideation, and psychosis. Participants rate each on a five-point scale from 0 (none) to 4 (very). In Najarian’s work (2001), the SCL-25 showed a good agreement with its parent, the SCL-90, suggesting that it can be used to evaluate symptoms of mental illness [23]. For the SCL-25 in our research, Cronbach’s alpha coefficient is.70, indicating good internal consistency and reliability.

***Demographic information questionnaire and caffeine consumption checklist*** is a researcher-developed questionnaire for the systematic collection of personal information, the description of caffeine usage, and measurement of caffeine consumption. Age (in years), sex (female or male), education (high school, bachelor’s degree, and master’s degree or higher), job (unemployed, employed, retired, and student), and relationship status (single, married, divorced, in a relationship, and widowed).

### Data screening

Before we started performing the analysis, detailed data screening processes were followed to verify the dataset’s quality. That meant inspecting all the variables for missing values, outliers, and data entry mistakes. Incomplete data were corrected with the proper imputation, and outliers were scrutinized for quality control. We also calculated descriptive statistics to give an overview of the data’s features and detect the anomalies. Any anomalies observed during the screening were identified and corrected so as to keep data consistent. To make the answers more accurate and reliable, only those respondents who answered over 10% of the questions were included. Altogether, 1304 people started out, and 1228 of those who met the requirements — after dropping respondents with randomly answered answers or incomplete answers. If you are interested in understanding more about data screening, check out the previous paper from this database, which tried to calculate the prevalence of Caffeine Use Disorder through very different methods and purposes [20].

### Analytic plan

#### Aims 1 and 2

To explore the network structure of CUD and caffeine withdrawal symptoms, we used R version 4.2.1 for network analysis (2022-06-23 ucrt, “Funny-Looking Kid”). The “bootnet” R package [24] generated this to create networks. For the estimate, we used the Gaussian graphical model. We computed the partial correlation matrix using the Extended Bayesian Information Criterion Graphical Least Absolute Shrinkage and Selection Operator (EBICglasso) algorithm to improve the network accuracy and readability [25]. They used the Bonferroni correction to eliminate false positives. We assigned symptoms to the nodes, and edge representations (1–1) described the associations between the nodes. R programs “graph” and “network tools” were used to find the node centrality indices such as the network’s strength, closeness, and betweenness. The expected impact was also given as a fourth centrality index to evaluate the expected importance of individual nodes across the whole network. Data was normalized to z-scores for a general comparison between different centrality indices. Bridge centrality was also used to determine whether certain nodes were influential as intermediaries or common symptoms among other clusters of symptoms.

#### Aim 3

To find bridge symptoms tying variables together in the network, we used the “MGM” package for multivariate Gaussian graphical modeling and the “EBICglasso” package for sparse network estimation [26]. In particular, EBIC with graphical LASSO penalty was used for network estimation, which allowed determining the relevant connections without biasing to false associations.

Subsequently, the “*bridge*” function from the “*MGM*” package was utilized to assess the Bridge Expected Influence (1-step) of individual symptoms [27]. This approach enables the characterization of symptoms that serve as crucial links between distinct communities or clusters within the network, shedding light on their potential role in facilitating interactions and information flow across the system.

## Results

### Research aim 1

The definition of the CUD and caffeine withdrawal symptoms is presented in Table 2. This table outlined nine distinct symptoms (according to CUDQ), each shedding light on different aspects of CUD, from the persistent desire to control caffeine use (symptoms one in DSM-5) to the presence of cravings (last symptom in DSM-5).

**Table 2:** Presenting CUD (CUD) and caffeine withdrawal (W) nodes and their definitions

Node	Heading	Definition
CUD.1	Desire or unsuccessful control	This criterion assesses the persistent desire to cut down or control caffeine use. In the network, it might be linked to other criteria, indicating its association with attempts to control caffeine consumption.
CUD.2	Continued Use despite problems	This criterion evaluates continued caffeine use despite knowing about physical or psychological problems attributed to caffeine. It could be central in the network, indicating its role in influencing other criteria.
CUD.3	Withdrawal symptoms	This criterion assesses the presence of withdrawal symptoms related to caffeine use. It may connect with other criteria, such as craving, or the amount of caffeine consumed.
CUD.4	Excessive consumption	This criterion relates to caffeine consumption in larger amounts or longer periods than intended. It may be linked to other criteria assessing the consequences of caffeine use.
CUD.5	Role obligations	This criterion evaluates whether caffeine use affects fulfilling major role obligations. In the network, it might indicate its impact on work, school, or home life.
CUD.6	Social/Interpersonal problems	This criterion looks at how caffeine use contributes to social or interpersonal problems. It could be connected to other criteria assessing the impact on relationships.
CUD.7	Tolerance	This criterion measures the development of tolerance, where more caffeine is needed to achieve the desired effect. It might be central in the network, connecting to other criteria.
CUD.8	Time spent on caffeine	This criterion examines the time spent on caffeine-related activities. In the network, it could be linked to criteria related to cravings or withdrawal symptoms.
CUD.9	Craving	This criterion assesses the presence of a craving or a strong desire to use caffeine. It may connect with other criteria, particularly those related to consumption and tolerance.
W1	Headache	Headache
W2	Fatigue	Marked fatigue or drowsiness
W3	Anxiety, depressed mood, or irritability	Dysphoric mood, depressed mood, or irritability
W4	Difficulty in Concentration	Difficulty concentrating
W5	Flu-like symptoms	Flu-like symptoms (nausea, vomiting, or muscle pain/stiffness)

**Fig. 1:**
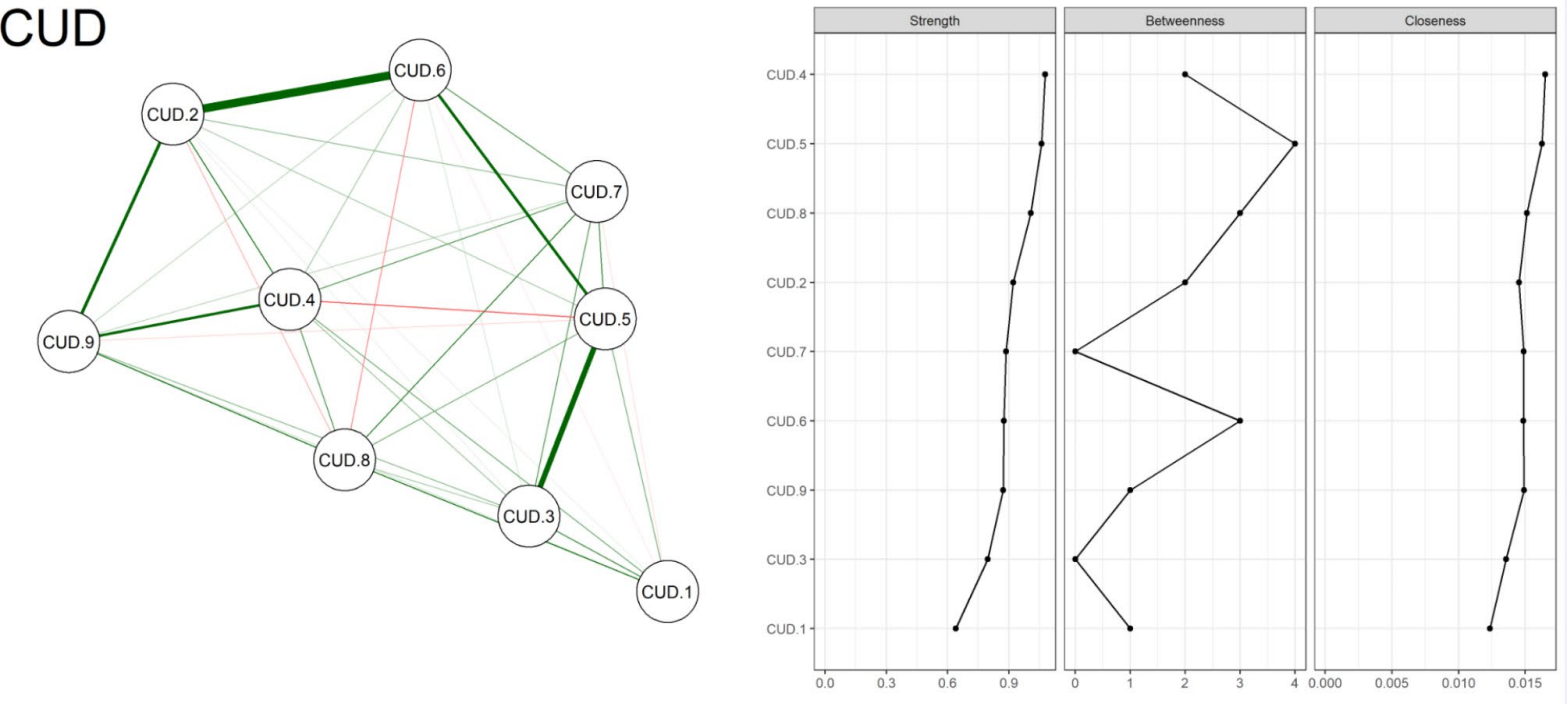
Network analysis of caffeine use disorder (left side) and its centrality measures (right side) (CUD.1: Desire or Unsuccessful Control, CUD.2 = Continued Use Despite Problems, CUD.3 = Withdrawal Symptoms, CUD.4 = Excessive Consumption, CUD.5 = Role Obligations, CUD.6 = Social/Interpersonal Problems CUD.7 = Tolerance, CUD.8 = Time Spent on Caffeine, CUD.9 = Craving)

Figure 1 (left side) presented the network analysis of CUD, visually representing the relationships between different criteria within the disorder. The figure highlighted the criteria’s interconnectedness and centrality within the CUD network, offering a comprehensive overview of the disorder’s dynamics. Figure 1 (right side) presented a graphical representation of centrality measures within the CUD network.

Figure 1 has shown centrality measures, including ‘strength,’ ‘betweenness,’ and ‘closeness,’ each of which quantifies the influence and significance of criteria in the network. Notably, the criteria are ordered by ‘Strength’ centrality, highlighting the most influential criteria in CUD. This visualization underscored the key nodes that play a pivotal role in shaping the CUD network and can serve as focal points for further analysis and understanding of the disorder’s dynamics. Please see Table 1 in the supplementary file for the numeric values of centrality measures.

In the context of a CUD, among the criteria assessed, node “*CUD.5*,” which represents recurrent caffeine use leading to a failure in fulfilling significant role obligations, emerges as a central and highly influential node within the network. This suggests that difficulties in meeting work, school, or home responsibilities due to caffeine use or withdrawal may profoundly impact the overall dynamics of CUD. Additionally, node “*CUD.4*”, indicating caffeine consumption in more significant amounts or overextended periods, exhibits substantial “*Strength*” centrality, signifying its pivotal role in the network. Conversely, nodes “*CUD.1*” and “*CUD.3*” appear to have lower centrality measures, suggesting they may have relatively less influence on the broader network dynamics.

### Research aim 2

**Fig. 2:**
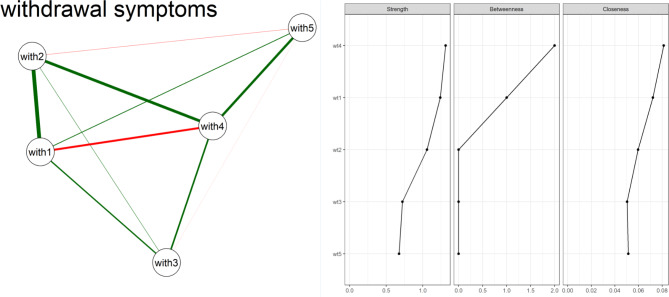
Network analysis of caffeine withdrawal (left side) and its centrality measures (right side) (W1 = Headache, W2 = Fatigue, W3 = Anxiety, depressed mood or irritability, W4 = Difficulty in Concentration, W5 = Flu-like symptoms)

In this study, we analyzed five caffeine withdrawal symptoms, including headache, fatigue or drowsiness, Anxiety, depressed mood or irritability, difficulty concentrating, and Flu-like symptoms. Figure 2 (left side) presents the network analysis of withdrawal symptoms, visually representing the relationships between different criteria within the disorder. The figure highlights the criteria’s interconnectedness and centrality within the withdrawal symptoms network, offering a comprehensive overview of the disorder’s dynamics. According to Fig. 2 and Table 2S, difficulty concentrating (W4) emerged as a central node within the network, indicating its strong connections with other symptoms. Conversely, Flu-like symptoms (W5) appeared to have fewer direct connections. This network perspective provides a deeper understanding of how these withdrawal symptoms relate to each other, which may have implications for treatment strategies and further research in this domain.

### Research aim 3

**Fig. 3:**
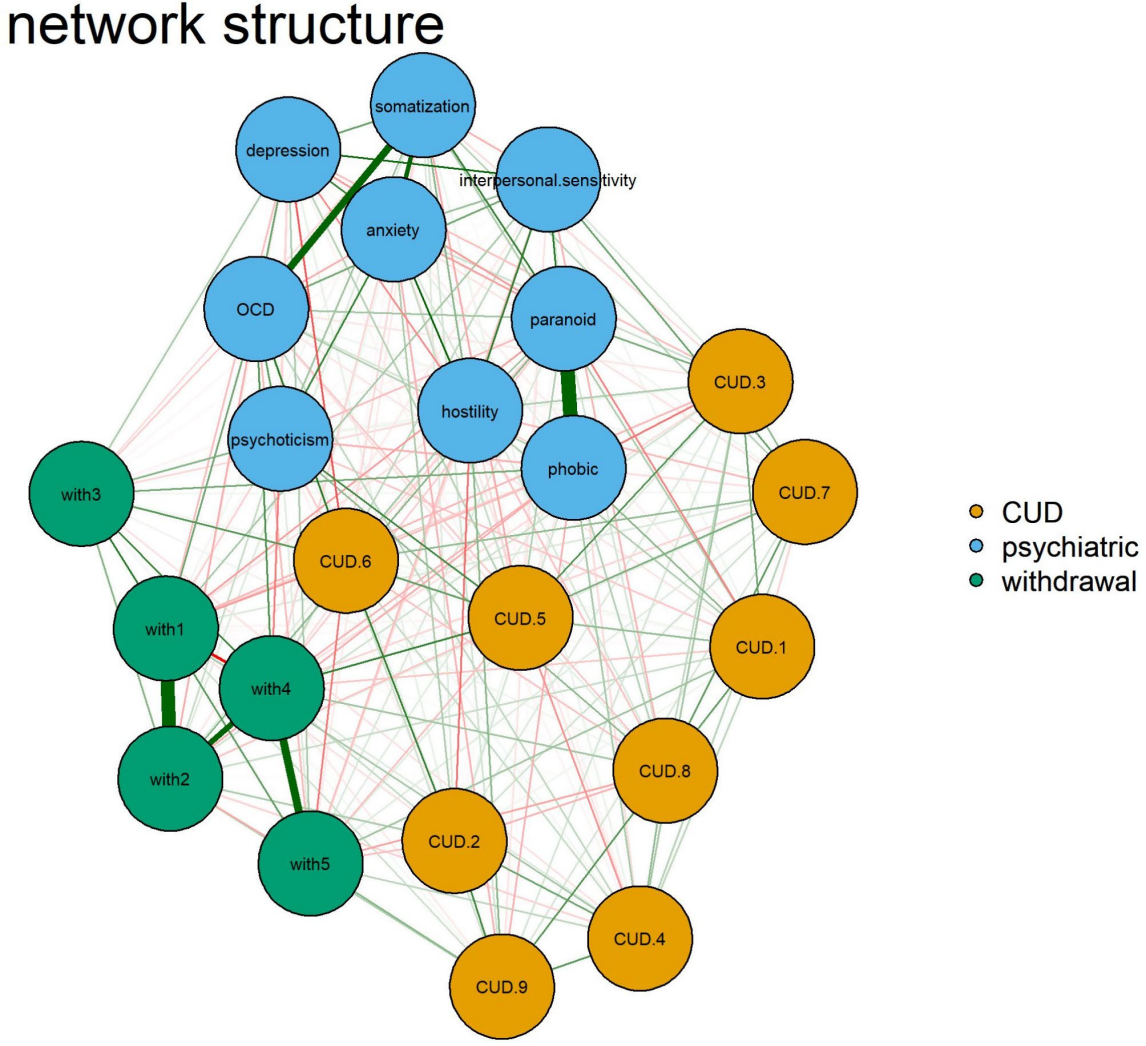
Interplay between caffeine use disorder, caffeine withdrawal, and psychiatric symptoms. [ CUD.1: Desire or Unsuccessful Control, CUD.2 = Continued Use Despite Problems, CUD.3 = Withdrawal Symptoms, CUD.4 = Excessive Consumption, CUD.5 = Role Obligations, CUD.6 = Social/Interpersonal Problems CUD.7 = Tolerance, CUD.8 = Time Spent on Caffeine, CUD.9 = Craving, W1 = Headache, W2 = Fatigue, W3 = Anxiety, depressed mood or irritability, W4 = Difficulty in Concentration, W5 = Flu-like symptoms, OCD = obsessive-compulsive disorder)

We used an extensive network analysis to examine how psychiatric symptoms, withdrawal symptoms, and CUD criteria related to each other. This analysis showed an emergent network architecture that revealed the complex relationships between these mental models and taught us about how they interact. Figure 3: The final network of symptoms Fig. 3The network of symptoms -End of Life. This network has nine nodes in blue, signifying psychiatric symptoms; nine in orange, representing CUD; and green nodes, indicating caffeine withdrawal symptoms. Notably, role obligations and social/interpersonal problems exhibit strong connections with both psychiatric symptoms and caffeine withdrawal symptoms.

**Fig. 4:**
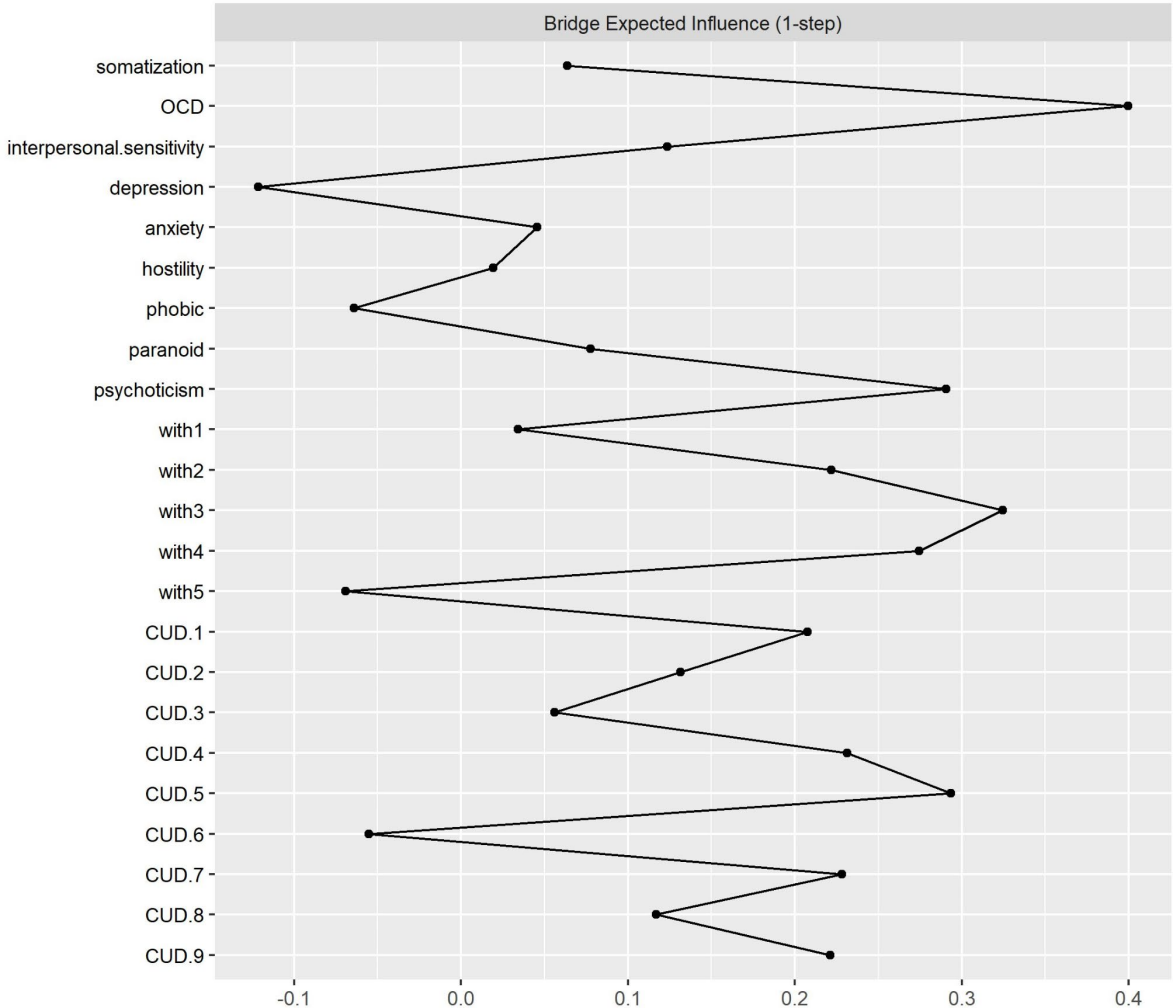
Bridge expected influence among variables. [ CUD.1: Desire or Unsuccessful Control, CUD.2 = Continued Use Despite Problems, CUD.3 = Withdrawal Symptoms, CUD.4 = Excessive Consumption, CUD.5 = Role Obligations, CUD.6 = Social/Interpersonal Problems CUD.7 = Tolerance, CUD.8 = Time Spent on Caffeine, CUD.9 = Craving, W1 = Headache, W2 = Fatigue, W3 = Anxiety, depressed mood or irritability, W4 = Difficulty in Concentration, W5 = Flu-like symptoms, OCD = obsessive-compulsive disorder)

Bridge Expected Influence was used to investigate the interaction between symptoms. Obsessive Compulsive Disorder (OCD) emerged as a central and highly influential node, indicating its pivotal role in the network. This finding underscores the potential importance of addressing CUD. Moreover, the positive influences of “*psychoticism*” and difficulty in concentration signify the interconnectedness of these factors in CUD. Notably, the strong influences of ‘craving’ and excessive consumption’ highlight their significance as critical contributors to the CUD construct. These insights contribute to understanding the complex relationships between psychological variables and have practical implications for assessing and treating individuals with CUD. Figure 4 and Table 3 presented these results.

**Table 3:** Bridge expected influence (1-step) results

Group	Node	Bridge Expected Influence (1-step)
Psychiatric symptoms	Somatization	.06
	Obsessive-compulsive disorder	.39
	Interpersonal sensitivity	.12
	Depression	− .12
	Anxiety	.04
	Hostility	.01
	Phobic	− .06
	Paranoid	.07
	Psychoticism	.29
	Headache	.03
	Fatigue	.22
Caffeine withdrawal	Anxiety, depressed mood or irritability	.32
	Difficulty in concentration	.27
	Flu-like symptoms	− .06
	desire or unsuccessful control	.20
	Continued use despite problems	.13
	Withdrawal symptoms	.05
	Excessive consumption	.26
CUD	Role obligations	.29
	Social/Interpersonal problems	− .05
	Tolerance	.22
	Time spent on caffeine	.12
	Craving	.22

## Discussion

Our main findings provide insight into the structural significance of specific symptoms within the CUD and caffeine withdrawal networks. Additionally, we estimated the pathways between caffeine-related symptoms and psychiatric symptoms using network analysis. It is important to note that when we refer to the ‘importance’ or ‘impact’ of symptoms in this context, we specifically reference statistical parameters, not necessarily clinical or theoretical significance unless explicitly stated otherwise.

First, we found ‘excessive consumption’ to be the most central and influential symptom in the CUD network. Second, ‘role obligations’ emerged as the second most significant node within the CUD structure. Conversely, ‘desire or unsuccessful control’ was identified as the network’s weakest and least influential symptom. To the best of our knowledge, no prior research has explored these relationships using this analytical approach. By examining the complex associations between symptoms, this study offers a novel perspective on the network structure of CUD, contributing to understanding its underlying dynamics and interactions. The importance of the " excessive consumption” node highlights the significance of consuming caffeine in more significant amounts or overextended periods, while the centrality of “*role obligations*” underscores the impact of recurrent caffeine use on fulfilling significant responsibilities. The centrality of these symptoms in the CUD network structure may be attributed to their substantial influence on various disorder dimensions. Excessive consumption, which focuses on the impact of caffeine use on fulfilling significant role obligations, is likely central due to its direct connection with crucial aspects of individuals’ daily functioning and responsibilities [28]. Difficulties in meeting work, school, or home obligations can have far-reaching consequences, significantly impacting individuals’ overall well-being and quality of life [28, 29].

Although the current study is the first to examine the network structure of the CUD, the findings are somewhat similar to those of previous studies. As mentioned earlier, the most important symptom in the network structure of CUD was excessive consumption. Previous studies have shown that excessive caffeine use is strongly associated with anxiety, sleep problems [30], depression [31], gastrointestinal disorders, tremors, and mental disorders [32]. Therefore, according to these studies, excessive caffeine use could be a significant factor in the context of CUD. Regarding caffeine withdrawal, ‘difficulty concentrating’ emerged as a central node within the network. As stated earlier, no similar study has compared its results with the present one. However, some research has shown similar results, indicating that difficulty in concentration can occur during the withdrawal of various substances [33, 34].

Based on our findings, the centrality of difficulty in concentration within caffeine withdrawal symptoms can potentially be attributed to several underlying factors. Firstly, the decreased concentration may result from heightened levels of anxiety and worry, which often accompany withdrawal symptoms from various substances [35, 36]. Secondly, somatic problems associated with withdrawal, such as physical discomfort and fatigue, may further contribute to impaired concentration [36]. Moreover, the physiological impact of substance withdrawal on cognitive processes could also play a significant role in this observed phenomenon. Also, this interplay of factors can make caffeine withdrawal even worse than difficulty concentrating [37, 38]. OCD was one of the network’s most potent and influencing nodes, which suggests that it had an essential influence on CUD. This result indicates that treating OCD symptoms may be critical in patients with CUD because OCD symptoms can intensify caffeine use and facilitate the persistence of distorted patterns of caffeine use. Because OCD is interwoven with other symptoms of the network, addressing OCD symptoms in treatments can have broad consequences for other symptoms of CUD [21]. Second, the “psychoticism” and focusing difficulty can be positive sources that further indicate the complicated relationship between many psychological variables and CUD. Psychoticism, or peculiar thinking, could lead to the onset or worsening of caffeine-related symptoms and possibly more severe cases of CUD [39]. Moreover, impaired concentration indicates the cognitive effects of caffeine consumption and withdrawal, so cognitive impairment could be an important factor in sustaining unhealthy caffeine habits [1].

And, of course, the centrality of ‘craving’ and binge-eating highlights their role as essential cogs in the CUD machine. Craving — cravings are excessive urges or desire to take caffeine — may encourage individuals to consume more than recommended even though it’s harmful, reinforcing the dependence loop [3]. Overconsumption, meanwhile, is the primary source of tolerance and withdrawal effects, which in turn keep addictive caffeine-related habits alive [2]. These underlying craving and overeating symptoms, therefore, need to be addressed when crafting interventions for CUD to interrupt the dependence process and promote more healthy consumption.

### Limitations and future directions

It is essential to acknowledge that this research possesses certain limitations. First, we used a Likert scale for the CUDQ, whereas the original version of this scale was based on a yes/no concept, in line with DSM-5 guidelines for diagnosis. However, as we needed a continuous range to estimate the network structure of CUD, the Likert scale was utilized. Thus, the first limitation is the lack of evaluation of the psychometric properties of the Likert version of CUDQ. Finally, the mean age of the participants in the current study (35.49 years) may limit the generalizability of our findings to older populations. Age-related differences in cognitive function, physical symptoms, and emotional responses could influence how caffeine use and withdrawal symptoms are experienced and reported. Future research might examine these factors across a broader age range to better understand potential variations. Also, future studies should focus on comparing the psychometric properties of both versions to provide a richer literature for evaluating caffeine-related problems. Secondly, the study’s participants were exclusively from Tehran, Iran, and as a result, the generalizability of the findings to other cities within Iran might be limited due to cultural differences. Subsequent research endeavors should strive to reproduce these results in more diverse samples. Lastly, caffeine consumption is a part of Iranian culture, so the level of CUD and caffeine withdrawal should be evaluated in different caffeine products.

### Research and clinical implications

The utilization of network analysis in studying CUD and withdrawal symptoms introduces a cutting-edge perspective, providing a comprehensive understanding of symptom interconnections. Future research avenues could explore comparative studies across diverse populations and cultures, aiming to replicate and generalize network structures. Longitudinal investigations may unveil the dynamic nature of caffeine-related symptoms over time. Clinically, recognizing the centrality of symptoms like “*excessive consumption*” and “*role obligations*” in CUD can guide targeted interventions for enhanced effectiveness. Addressing the central role of “*difficulty Concentrating*” during withdrawal highlights the need to tailor interventions to alleviate cognitive symptoms. Integrated treatment approaches considering the interconnectedness of psychiatric symptoms, withdrawal, and CUD criteria can optimize clinical outcomes. Cultural considerations are crucial, emphasizing tailoring interventions to diverse cultural contexts. Additionally, insights from network analysis can inform early identification and prevention efforts, facilitating proactive interventions for individuals at risk of developing problematic caffeine-related behaviors.

## Conclusion

This study utilized network analysis to explore the connections between CUD, caffeine withdrawal, and psychiatric symptoms within the general Iranian population. The findings underscored the importance of fulfilling significant role obligations and experiencing difficulty concentrating as key symptoms of CUD and caffeine withdrawal, respectively. Particularly noteworthy was the prominence of OCD as a highly influential node, emphasizing the importance of addressing OCD symptoms among individuals diagnosed with CUD.

## Electronic supplementary material

Below is the link to the electronic supplementary material.

Supplementary Material 1

## Data Availability

The raw data supporting the conclusions of the present research is available at https://osf.io/gntrx.
