# Supplementary material for: Network analysis of caffeine use disorder, withdrawal symptoms, and psychiatric symptoms
Source: BMC Psychiatry. 2025 Jan 21;25:66. doi: 10.1186/s12888-025-06478-z (PMC11753023; doi:10.1186/s12888-025-06478-z)
Supplement: Supplementary file 1 — Supplementary Material 1 [file 12888_2025_6478_MOESM1_ESM.docx]

Table.1S: Centrality Measures for Caffeine Use Disorder (CUD) Network

| **Node** | **Betweenness** | **Closeness** | **Strength** | **ExpectedInfluence** |
| --- | --- | --- | --- | --- |
| CUD.1 | -0.55777335 | -1.91711185 | -1.94110586 | -2.03823489 |
| CUD.2 | 0.15936381 | -0.20142293 | 0.13002264 | 0.71774186 |
| CUD.3 | -1.27491052 | -0.96084439 | -0.78394600 | 0.59842904 |
| CUD.4 | 0.15936381 | 1.37201381 | 1.26569927 | 0.44184087 |
| CUD.5 | 1.59363815 | 1.17407578 | 1.14873416 | -0.42631832 |
| CUD.6 | 0.87650098 | 0.07481439 | -0.21034663 | -0.83476816 |
| CUD.7 | -1.27491052 | 0.07848185 | -0.12926134 | 1.09467077 |
| CUD.8 | 0.87650098 | 0.27960444 | 0.75141942 | -0.32461859 |
| CUD.9 | -0.55777335 | 0.10038890 | -0.23121566 | 0.77125740 |

| Table.2S: Centrality Measures for caffeine withdrawal symptoms Network | | | | |
| --- | --- | --- | --- | --- |
| **Node** | **Betweenness** | **Closeness** | **Strength** | **ExpectedInfluence** |
| W.1 | 0.447214 | 0.681467 | 0.813416 | -0.32472 |
| W.2 | -0.67082 | -0.2472 | 0.196307 | 1.407044 |
| W.3 | -0.67082 | -0.93365 | -0.96045 | 0.132768 |
| W.4 | 1.565248 | 1.362817 | 1.06535 | 0.153683 |
| W.5 | -0.67082 | -0.86343 | -1.11463 | -1.36877 |
